# Neighbourhood ethnic density and psychosis — Is there a difference according to generation?

**DOI:** 10.1016/j.schres.2017.09.029

**Published:** 2018-05

**Authors:** Peter Schofield, Malene Thygesen, Jayati Das-Munshi, Laia Becares, Elizabeth Cantor-Graae, Esben Agerbo, Carsten Pedersen

**Affiliations:** a Division of Health & Social Care Research, Faculty of Life Sciences & Medicine, King's College London, London, United Kingdom; b Health Service & Population Research Department, Institute of Psychiatry, Psychology & Neuroscience, King's College London, London, United Kingdom; c Lundbeck Foundation Initiative for Integrative Psychiatric Research, iPSYCH, Aarhus, Denmark; d National Centre for Register-Based Research, Aarhus University, Aarhus, Denmark; e CIRRAU — Centre for Integrated Register-based Research at Aarhus University, Aarhus, Denmark; f Centre on Dynamics of Ethnicity, The University of Manchester, Manchester, UK; g Social Medicine and Global Health, Lund University, Lund, Sweden

**Keywords:** Aetiology, Social determinants, Ethnicity

## Abstract

**Background:**

For different migrant groups living in an area with few people from the same ethnic background is associated with increased psychosis incidence (the ethnic density effect). We set out to answer the question: are there generational differences in this effect?

**Methods:**

Analysis of a population based cohort (2.2 million) comprising all those born 1st January 1965, or later, living in Denmark on their 15th birthday. This included 90,476 migrants from Africa, Europe (excluding Scandinavia) and the Middle East, with 55% first generation and the rest second-generation migrants. Neighbourhood co-ethnic density was determined at age 15 and we adjusted for age, gender, calendar period, parental psychiatric history and parental income.

**Results:**

For first-generation migrants from Africa, there was no statistically significant difference (*p* = 0.30) in psychosis rates when comparing lowest with highest ethnic density quintiles, whereas the second generation showed a 3.87-fold (95% CI 1.77–8.48) increase. Similarly, for migrants from the Middle East, the first generation showed no evidence of an ethnic density effect (*p* = 0.94) while the second showed a clear increase in psychosis when comparing lowest with highest quintiles, incidence rate ratio (IRR) 2.43 (95% CI, 1.18–5.00). For European migrants, there was some limited evidence of an effect in the first generation, (IRR) 1.69 (95% CI, 1.19–2.40), with this slightly raised in the second: IRR 1.80 (95% CI, 1.27–2.56).

**Conclusions:**

We found strong evidence for an ethnic density effect on psychosis incidence for second-generation migrants but this was either weak or absent for the first generation.

## 1 Introduction

Migrant groups are consistently shown to have an increased risk of psychotic illness which persists from one generation to the next (Bourque et al., 2011, Cantor-Graae and Pedersen, 2013, Cantor-Graae and Selten, 2005). In recent years, therefore, much research attention has been paid to the post-migration social environment, and it has been repeatedly shown that living in a low ‘ethnic density’ area (with few people from the corresponding ethnic group) is associated with increased psychosis incidence (Boydell et al., 2001, Kirkbride et al., 2007a; Schofield et al., 2011a, Schofield et al., 2011b, Veling et al., 2008). However, it is not known how this might contribute to the increased risk persisting from one generation to the next.

The ethnic density effect has been linked to both the process of acculturation, the meeting of migrant and host cultures and the consequent psychological stress, and also the experience of discrimination (Becares et al., 2009, Halpern and Nazroo, 2000, Jurcik et al., 2013, Shaw et al., 2012). Both factors, it is argued, could be more salient for the second generation (Mahy et al., 1999, McIntyre et al., 2016, Nakash et al., 2012, Smith et al., 2009, Williams et al., 2007). Studies of generational differences have an important role to play generally in helping us understand the increased risk of psychosis among migrant groups (Bourque et al., 2011). Therefore, investigating generational differences in the effect of neighbourhood ethnic density could help further our understanding of processes behind this, as yet, little understood risk factor for psychosis.

However, to date no studies have addressed this question. This is perhaps not surprising given the inherent sample size problems when investigating members of minority ethnic groups in areas where their ethnic group is under-represented. A further problem is differential exposure where the exposure period is likely shorter for the first generation compared to those born in the host country. One solution would be to use a whole population cohort design ensuring the first generation has a minimum exposure period.

This is the first nationwide population based study that sets out to disentangle the effect of ethnic density between first and second generation migrants. We could achieve this using whole population cohort data covering migrants to Denmark for a period of up to thirty years or more, linked to information about their neighbourhood at age 15. In this way, we set out to answer the question: are there generational differences in the association between ethnic density at age 15 and later incidence of non-affective psychosis?”

## 2 Method

### 2.1 Sample

We used data from the Danish Civil Registration System, including demographic details and links to parental data and place of residence as well as a unique personal identification number allowing data to be linked across population registers (Pedersen et al., 2006). We followed all those born after 1st January 1965 and living in Denmark on their 15th birthday until they either died, migrated, were diagnosed with a non-affective psychotic illness or 1st of July 2013 (whichever came first). Further details on this cohort are reported in a previously published study (Schofield et al., 2017).

### 2.2 Measures

We linked this to the Danish Psychiatric Central Register (Munk-Jørgensen and Mortensen, 1997) which covers all psychiatric in-patient admissions and also, from 1995, all psychiatric out-patient visits. Non-affective psychosis was defined as ICD-10 codes F20–F29 and their ICD-8 equivalents (ICD-8 295.x9, 296.89, 297.x9, 298.29-298.99, 299.04, 299.05, 299.09, 301.83) based on clinical diagnoses assigned at discharge. Date of onset was defined as the first day of first contact with this diagnosis, and we excluded anyone with a diagnosis prior to their 15th birthday.

Ethnic group is not recorded in Danish registry data therefore we use, as a proxy, region of origin based on the cohort members' country of birth as well as their parents' country of birth and date of migration. This is in line with previous studies using Danish register data (Schofield et al., 2017, Cantor-Graae et al., 2003, Cantor-Graae and Pedersen, 2007). Similarly, at an area level, as a proxy for own-group ethnic density we use own-migrant group density based on the proportion of people who were born, or whose parents were born, in the same region as the defined migrant group. We categorised region of origin as Africa, Europe (not Scandinavia) and the Middle East as previously (Cantor-Graae et al., 2003, Schofield et al., 2017). First generation migrants were designated as persons born abroad and whose parents were also born abroad; all in the same region. We incorporated *both* parents' birth region as this has been shown most relevant to psychosis risk (Cantor-Graae and Pedersen, 2007). We therefore excluded instances where parents' birth region differed.

Parental country of birth was missing, for at least one parent, for 23% of those born in the regions we looked at. In these instances, we assumed missing parental birth region was the same as that of the cohort member. We could assume parents with missing data were not born in Denmark as this would have been recorded (Pedersen et al., 2006). Their birth region could still differ from the cohort member although this was rare among those for whom we did have parental data (3%) and could therefore be discounted.

Second generation migrants were designated as those born in Denmark but with both parents born outside of Denmark. Region of origin for this group was based on the birth region of both parents and, again, we excluded instances where parents were born in different regions. For the second generation, because region of origin was entirely determined by parental place of birth we also excluded anyone with missing parental data (1.4%).

#### 2.2.1 Neighbourhood level measures

These were based on Danish parishes, originally derived from ecclesiastical boundaries dating back to the middle ages, which continue to play a role in demarcating communities and school districts (OECD, 2016). These were adapted to make units more homogenous in size, as we describe in more detail in our previous study, resulting in a total of 1167 parish units with a median size of 3564 people (Schofield et al., 2017).

For each parish and migrant region, neighbourhood ethnic density was defined as the proportion of all migrants from that region living in the parish in the year the cohort member was 15, divided into quintiles. We chose neighbourhood at age 15 to reflect the childhood social environment, at a point when residential history would most likely be stable and to maximise the sample size. We used the definition of migrant groups outlined above but combining both first and second generations. We had complete reference to both parents for all those born in Denmark in 1960 or later (Pedersen et al., 2006). Because immigration into Denmark was very low prior to 1960, mainly from adjoining countries (Nannestad, 2004), we therefore assumed parish members born in Denmark with missing parental data were Danish.

#### 2.2.2 Exclusions

We excluded all foreign born adoptees to avoid confounding where this group might be at a higher risk of psychosis and more likely to live in low ethnic density areas (Cantor-Graae and Pedersen, 2013). We defined these as anyone born outside Denmark where both (legal) parents were born in Denmark (1.28% of the cohort).

#### 2.2.3 Parental information

To account for possible confounding where parental mental illness influences the neighbourhood where cohort members live at age 15 we adjusted for any record of a psychiatric disorder in either parent (Dean et al., 2010). We also adjusted for parental socio-economic background based on combined parental gross annual income when the child was aged 15. Where father's income was missing and the mother was categorised as a single parent we used mother's income only.

### 2.3 Statistical analysis

We used multilevel Poisson regression to model effects at: 1) individual, 2) year (in which aged 15) and 3) neighbourhood (parish) levels. The effect of ethnic density on psychosis incidence was modelled as a cross-level interaction between migrant group, neighbourhood co-ethnic density at age 15 and generational status (first or second generation). We went on to assess the overall linear trend by entering ethnic density quintiles as a continuous variable.

All analyses were adjusted for age, gender (and their interaction), calendar time, and a history of parental psychiatric disorder. Age and calendar time were included as time varying covariates (Clayton et al., 1993) with age categorised using the following cut-off points: 15, 20, 25, 30, 35, 40, 45, 50, and 55 or older and calendar time using 5 year bands, except for the 1990s where 2-year bands were used to account for changes to the ICD system.

We also allowed for over-dispersion using negative binomial regression. This made no difference to the main study results and therefore only the Poisson model results are presented here.

All analyses were conducted using Stata version 14 (StataCorp, 2015).

### 2.4 Sensitivity analysis

To account for between generation differences in duration of neighbourhood exposure we repeated the analysis for the first generation but restricting the cohort to those living in Denmark for at least 7 years. We chose this time-period to maximise exposure time while retaining a sample comparable in size to the second generation. Also, to ensure that our results overall were invariant to the way we chose to define ethnic density we re-ran the analysis using a continuous ethnic density measure and compared this with the main analysis using ethnic density quintiles.

### 2.5 Ethical approval

The study was approved by the Danish Data Protection Agency.

## 3 Results

### 3.1 Sample

We followed 2,195,684 Danish citizens over 31,525,426 person years. This included 49,606 first generation migrants, from the defined regions, and 40,870 of the second generation. Of these, 1230 first generation and 592 second generation migrants were diagnosed with a non-affective psychosis during follow-up. Incidence was raised in the first generation compared with the second for each group (see Table 1). In comparison the crude psychosis incidence rate for native Danes was 7.2 (95% CI 7.1–7.3) cases per 10,000 person years.

**Table 1:** Incidence of non-affective psychosis by migrant group — first and second generation migrants compared to native Danes.

Migrant group (country of origin)^a^	1st generation					2nd generation				
	Total (N)	Person-years	Cases	Crude incidence rate^b^	Incidence rate ratio (95% CI)^c^	Total (N)	Person-years	Cases	Crude incidence rate^b^	Incidence rate ratio (95% CI)^c^
Africa	7187	71,419	236	31.0	3.25 (2.89–3.66)	4593	37,162	80	21.5	2.11 (1.69–2.63)
Europe (non-Scandinavian)	24,436	345,911	585	16.9	2.01 (1.87–2.16)	25,984	254,743	410	16.1	1.63 (1.47–1.79)
Middle East	17,983	182,691	412	22.6	2.15 (1.95–2.36)	10,293	55,730	102	18.3	1.64 (1.35–1.99)

a Migrant group, for the first generation, is based on country of birth of cohort member and both parents and for the second generation, born in Denmark, the country of birth of both parents.

b The incidence rate measures the number of new cases per 10,000 person years at risk.

c Incidence rate ratios compare incidence of psychosis with native Danes adjusted for age, gender and calendar period.

### 3.2 Ethnic density associations compared across generations

The association between neighbourhood ethnic density and later psychosis incidence was clear for second generation migrants in our study and either absent, or reduced, for the first generation (Table 2). For the first-generation African group, there was no statistically significant difference in psychosis rates (*p* = 0.30), when comparing the lowest with the highest ethnic density quintiles, whereas for the second generation there was a 3.87-fold (95% CI 1.77–8.48) increased incidence. Similarly, for the Middle East group there was no statistically significant difference (*p* = 0.94) for the first generation while the second generation showed a clear increase in psychosis rates, incidence rate ratio (IRR) 2.43 (95% CI, 1.18–5.00), when comparing lowest and highest quintiles. For the group from Europe (non-Scandinavian) there was some evidence of an ethnic density effect in both the first generation, (IRR) 1.69 (95% CI, 1.19–2.40), comparing lowest and highest quintiles, and the second generation: IRR 1.13 (95% CI, 1.05–1.22). We also looked at the overall linear trend (Table 2), in terms of the average increase in incidence associated with a one quintile increase in ethnic density. For the first generation, European group this showed no statistically significant difference, (IRR) 1.06 (95% CI, 0.99 to 1.14, *p* = 0.12), and a linear trend was only apparent among the second generation, (IRR) 1.13 (95% CI, 1.05 to 1.22). The same pattern emerged for the other groups, with the African group showing a weak, non-statistically significant (*p* = 0.18), trend for the first generation, (IRR) 1.09 (95% CI, 0.96 to 1.23), in contrast to a clear linear trend for the second generation, (IRR) 1.43 (95% CI, 1.19 to 1.72). For the Middle East group, there was, again, no evidence of a linear trend for the first generation, (IRR) 0.98 (95% CI, 0.91 to 1.06, *p* = 0.66), in contrast to the second, (IRR) 1.24 (95% CI, 1.07 to 1.44).

**Table 2:** Incidence rate ratios of non-affective psychosis by neighbourhood ethnic density at age 15 for each migrant group – first and second generation compared.

		1st generation		2nd generation		1st generation	2nd generation
Ethnic density (quintiles for each group)	Ethnic density (%)	Cases	Crude Incidence Rate^a^	Cases	Crude Incidence Rate^a^	Incidence rate ratio (95% CI)^b^	Incidence rate ratio (95% CI)^b^
Africa							
1 (lowest)	< 0.4	34	33.8	11	53.1	1.39 (0.75–2.57)	3.87 (1.77–8.48)
2	0.4–0.9	73	38.6	18	32.8	1.60 (0.97–2.62)	2.31 (1.16–4.61)
3	0.9–1.7	41	26.1	16	18.7	0.92 (0.53–1.61)	1.04 (0.51–2.14)
4	1.7–3.7	54	35.8	20	15.3	1.35 (0.81–2.26)	0.90 (0.46–1.76)
5 (highest)	3.7–18.5	33	28.5	15	18.7	1	1
*Ethnic density trend*^c^						*1.09 (0.96–1.23) [p* *=* *0.18]*	*1.43 (1.19–1.72) [p* *<* *0.001]*
*Comparing ethnic density trend across generations*^d^						*1.33 (1.07–1.64) [p* *=* *0.01]*	

Europe							
1 (lowest)	< 2.3	140	18.3	52	18.6	1.69 (1.19–2.40)	1.80 (1.27–2.56)
2	2.3–3.9	125	15.1	69	16.5	1.41 (0.99–2.00)	1.47 (1.08–2.02)
3	3.9–5.9	126	17.0	88	15.8	1.32 (0.93–1.88)	1.22 (0.91–1.63)
4	5.9–9.4	140	20.0	102	17.5	1.66 (1.18–2.34)	1.18 (0.89–1.57)
5 (highest)	9.4–26.4	54	12.8	99	14.0	1	1
*Ethnic density trend*^c^						*1.06 (0.99–1.14) [p* *=* *0.10]*	*1.13 (1.05–1.22) [p* *=* *0.001]*
*Comparing ethnic density trend across generations*^d^						*1.08 (0.98–1.19) [p* *=* *0.12]*	

Middle East							
1 (lowest)	< 0.8	94	24.3	13	21.5	1.01 (0.72–1.42)	2.43 (1.18–5.00)
2	0.8–1.7	82	19.5	19	25.7	0.78 (0.56–1.10)	2.60 (1.38–4.90)
3	1.7–3.3	71	19.0	20	18.4	0.74 (0.52–1.06)	1.53 (0.80–2.92)
4	3.3–6.7	79	24.4	29	20.3	0.92 (0.65–1.30)	1.84 (1.03–3.29)
5 (highest)	6.7–40.0	84	26.3	21	12.3	1	1
*Ethnic density trend*^c^						0.98 (0.91–1.06) [*p* = 0.66]	1.24 (1.07–1.44) [*p* = 0.004]
*Comparing ethnic density trend across generations*^d^						*1.25 (1.06–1.47) [p* *=* *0.007]*	

a The incidence rate measures the number of new cases per 10,000 person years at risk.

b Adjusted for age, gender, calendar period, parental psychiatric history and income at age 15 the IRR compares rates at each level of ethnic density with the highest ethnic density quintile for that migrant group.

c Trend shows the incidence rate ratio corresponding to one quintile increase in neighbourhood ethnic density at age 15.

d Incidence rate ratio comparing overall trend for 2nd generation with the trend for the 1st generation.

We also re-analysed the data restricting first generation migrants to those who had been living in Denmark for at least 7 years prior to their 15th birthday. This made little difference to the main analysis results (Appendix Table 3) other than to further accentuate the between generation differences that we have already described. Also, when we re-ran the main analysis using a continuous measure of ethnic density a very similar pattern emerged as with the quintiles based analysis (Appendix Table 4).

Lastly, these results describe relative differences within each of the migrant groups we looked at. We also present the same results but this time comparing each group with native Danes. Here the reference group is native Danes in the least ethnically dense quintile. (Appendix Table 5). Here it is apparent that any increased risk of psychosis among migrants is no longer statistically significant for the second generation living in high ethnic density areas, and for the Middle East group there is actually a lower than average risk, (IRR) 0.58 (95% CI, 0.37–0.91). Conversely, first generation African and Middle Eastern migrants in high ethnic density areas continue to be at an increased risk of psychosis.

## 4 Discussion

### 4.1 Summary of the results

We found that associations between neighbourhood ethnic density and later non-affective psychosis were largely confined to second generation migrants. This was most apparent for those originating from Africa and the Middle East. Among the first generation, only the group originating from Europe showed evidence of any ethnic density effect (although even here the overall trend was not statistically significant).

### 4.2 Strengths and limitations

This is the first study to compare across generations the association between psychosis and neighbourhood ethnic density, taking advantage of a population cohort design where neighbourhood exposure is determined well in advance of illness onset. The register data, on which this was based, comprises all Danish residents and contains information on place of residence for almost everyone (99.7%) (Pedersen et al., 2006).

There are, though, some limitations to note: firstly, caution is needed when making comparisons between region of origin categories as these are far from homogenous, sometimes incorporating disparate ethnic groups with different migration experiences. We cannot, therefore, rule out the possibility that cross-generational differences may relate to different countries of origin from within these broad regions. However, the fact that cross-generational differences are so consistent suggests this alone is unlikely to explain the results we found. Secondly, the study is reliant on clinical data only for diagnosis and therefore potentially subject to bias due to differential service use. It is possible that migrant groups may be less inclined to engage with mental health services than the Danish population and this could be more likely in areas where migrants are more concentrated. However, if this were the case we would expect first generation migrants, compared to the second, to be both less likely to contact services and more inclined to resist service use in high ethnic density areas. In fact, we find neither: first generation migrants were more likely to be diagnosed with psychosis compared to the second generation and less likely to demonstrate any corresponding ethnic density effect. Therefore, it appears that differential service use is unlikely to explain the different ethnic density effects we have reported here.

### 4.3 Comparison with previous studies

Our initial analysis showed a higher rate of psychosis among first compared to second generation migrants (Table 1). A recent review reports no statistical significant difference in psychosis risk between generations (Bourque et al., 2011). However, our study showing a greater risk among the first generation is in line with other Danish population studies (Cantor-Graae et al., 2003, Cantor-Graae and Pedersen, 2013).

The ethnic density effect we report for second generation migrants are in line with previous ethnic density studies where generations have been conflated (Boydell et al., 2001, Kirkbride et al., 2014, Kirkbride et al., 2007b, Schofield et al., 2011a, Schofield et al., 2011b, Veling et al., 2008). Typically, studies have shown up to double the incidence of psychosis in low versus high ethnic density areas. The comparably larger effect sizes we have shown, particularly for second generation Africans, may be because previous studies miss-attributed ethnic density effects to the first generation.

### 4.4 Interpretation

By distinguishing between generations our results point to different causal processes behind the increased risk of psychosis among migrants. For the first generation, these may be more directly related to the migration process and, for refugees and forced migrants, experience of trauma in the country of origin. For the second generation, this is more likely related to the social context in which they are now living in Denmark. In fact, while migrants are at an overall increased risk of psychosis compared with native Danes, for the second generation in high ethnic density areas this difference is no longer statistically significant (Appendix Table 5).

The importance of social context for the second generation may be attributed to the stress of acculturation due to both marginalisation and assimilation. Marginalisation, a failure to identify with either the country of origin or the host country, is regarded as the least adaptive outcome of the acculturation process and the most likely to lead to mental ill health (Berry, 2005, Berry et al., 1987, Park, 1928). This has been linked with the ethnic density effect with those in low ethnic density areas more prone to experience marginalisation (Shaw et al., 2012). Poor mental health due to marginalised status may, in turn, be a feature of the 2nd generation; caught between their parents' culture, from which they feel disconnected, and the host culture, with which they cannot identify (Bhugra et al., 1999, McIntyre et al., 2016, Williams et al., 2007). A second outcome of the acculturation process, assimilation, describes relinquishing the culture of origin to become subsumed in the host culture (Berry, 1997). Again, this is associated with a greater risk of mental health problems and may be a more likely outcome for the second generation in areas where there are few people with their parents' cultural background. It has also been proposed that discrimination could be an underlying factor; with migrants living in higher ethnic density areas subject to lower levels of discrimination (Becares et al., 2009, Halpern and Nazroo, 2000). Again, it is argued, this may be modified by generational status where the second generation could be more vulnerable to the impact of discrimination on their self-esteem and more likely to perceive discrimination as a threat to their identity (Nakash et al., 2012, Smith et al., 2009). It is notable that migrants from elsewhere in Europe show the lowest ethnic density effect, barely modified by generational status, and this may reflect both a less stressful acculturation process for this group and lower perceived discrimination. It is also possible that factors more specific to the Danish context may be relevant. For example, in Denmark there is a very strong emphasis on integrating migrants into Danish society which, some have argued (Lindley and Van Hear, 2007, Valentine et al., 2009), could intensify any potential conflict between the culture of origin and the host culture.

### 4.5 Implications

Could it be that the ethnic density effect, long established in aetiological research, is largely experienced by second generation migrants alone? This has important implications for the likely mechanism behind these effects and our understanding of how the increased risk of psychosis persists from one generation to the next. Therefore, further study is needed to address these questions in other international contexts.

## Contributors

PS was responsible for the initial study design, analysis and interpretation of the study and drafting of the manuscript. LB, JD, EA, CP and MT were all involved in study design and interpretation of results. All authors contributed to and have approved the final manuscript.

## Conflict of interest

The study authors have nothing to disclose.

## Funding

This work was supported by a UK Medical Research Council fellowship (MR/K021494/1) to P.S.; a Hallsworth Research Fellowship and a UK Economic and Social Research Council Future Research Leaders grant (ES/K001582/1) to L.B.; Aarhus University, the Lundbeck Foundation, the Stanley Medical Research Institute to both E.A. and C.P.; and the Health Foundation working with the Academy of Medical Sciences to J.D.

## Role of funding source

The funding source had no role in the study design, in the collection, analysis and interpretation of data, in the writing of the manuscript and in the decision to submit the article for publication.

## Appendix A Supplementary data

Supplementary tables Image 1
