# Supplementary material for: Neighbourhood ethnic density and psychosis — Is there a difference according to generation?
Source: Schizophr Res. 2018 May;195:501–5. doi: 10.1016/j.schres.2017.09.029 (PMC5889713; doi:10.1016/j.schres.2017.09.029)
Supplement: Supplementary file 1 — Supplementary tables [file mmc1.docx]

## Appendix

Table 3 Incidence rate ratios of non-affective psychosis by neighbourhood ethnic density at age 15 for each migrant group – first generation migrants living in Denmark for at least 7 years

| Ethnic density (quintiles for each group) | Ethnic density (%) | Cases | Crude Incidence Rate^a^ | Incidence rate ratio (95% CI)^b^ |
| --- | --- | --- | --- | --- |
| Africa |  |  |  |  |
| 1 (lowest) | <0.4 | 9 | 24.9 | 1.14 (0.40 - 3.26) |
| 2 | 0.4 - 0.9 | 20 | 30.5 | 1.26 (0.57 - 2.78) |
| 3 | 0.9 - 1.7 | 10 | 14.7 | 0.73 (0.31 - 1.74) |
| 4 | 1.7 - 3.7 | 19 | 29.9 | 1.35 (0.64 - 2.85) |
| 5 (highest) | 3.7 - 18.5 | 18 | 32.8 | 1 |
| *Ethnic density trend*^c^ | |  | *1.01 (0.82 – 1.24)* | |
|  |  |  |  |  |
| Europe |  |  |  |  |
| 1 (lowest) | <2.3 | 56 | 3.4 | 1.58 (1.00 - 2.48) |
| 2 | 2.3 - 3.9 | 60 | 3.9 | 1.31 (0.84 - 2.06) |
| 3 | 3.9 - 5.9 | 59 | 3.6 | 1.13 (0.72 - 1.77) |
| 4 | 5.9 - 9.4 | 81 | 3.5 | 1.65 (1.08 - 2.52) |
| 5 (highest) | 9.4 - 26.4 | 35 | 2.4 | 1 |
| *Ethnic density trend*^c^ | |  | *1.04 (0.95 – 1.15)* | |
|  |  |  |  |  |
| Middle East |  |  |  |  |
| 1 (lowest) | <0.8 | 30 | 1.4 | 0.81 (0.49 - 1.34) |
| 2 | 0.8 - 1.7 | 33 | 1.6 | 0.74 (0.46 - 1.19) |
| 3 | 1.7 - 3.3 | 40 | 1.8 | 0.85 (0.55 - 1.31) |
| 4 | 3.3 - 6.7 | 52 | 1.9 | 0.90 (0.59 - 1.37) |
| 5 (highest) | 6.7 - 40.0 | 54 | 2.0 | 1 |
| *Ethnic density trend*^c^ | |  | 0.93 (0.83 – 1.04) | |

^a^The incidence rate measures the number of new cases per 10,000 person years at risk
^b^Adjusted for age, gender, calendar period, parental psychiatric history and income at age 15 the IRR compares rates at each level of ethnic density with the highest ethnic density quintile for that migrant group.
^c^Trend shows the incidence rate ratio corresponding to one quintile increase in neighbourhood ethnic density at age 15

Table 4 Incidence rate ratios of non-affective psychosis by neighbourhood ethnic density at age 15 for each migrant group – first and second generation compared using a continuous measure of ethnic density

|  | 1st generation | 2nd generation |
| --- | --- | --- |
| Ethnic density (quintiles for each group) | Incidence rate ratio (95% CI)^b^ | Incidence rate ratio (95% CI)^b^ |
| Africa |  |  |
| Effect of 10% decrease in ethnic density^a^ | 1.94 (0.84 - 4.52) | 6.27 (1.46 - 26.90) |
| Effect compared between generations | 3.23 (0.60 - 17.21) | |
| Europe |  |  |
| Effect of 10% decrease in ethnic density^a^ | 1.30 (0.99 - 1.70) | 1.31 (1.02 - 1.67) |
| Effect compared between generations | 1.00 (0.70 - 1.43) | |
| Middle East |  |  |
| Effect of 10% decrease in ethnic density^a^ | 0.99 (0.74 - 1.34) | 1.70 (1.04 - 2.77) |
| Effect compared between generations | 1.41 (1.11 - 1.80) | |

^a^Adjusted for age, gender, and calendar period the IRR shows the change in rate of non-affective psychosis corresponding
to a decrease of 10% in the ethnic density of the neighbourhood at age 15

Table 5 Incidence rate ratios of non-affective psychosis by neighbourhood ethnic density at age 15 for each migrant group – first and second generation compared – with reference to native Danes in lowest ethnic density quintile

|  |  | 1st generation | | 2nd generation | | 1st generation | 2nd generation |
| --- | --- | --- | --- | --- | --- | --- | --- |
| Ethnic density (quintiles for each group) | Ethnic density (%) | Cases | Crude Incidence Rate^a^ | Cases | Crude Incidence Rate^a^ | Incidence rate ratio (95% CI)^b^ | Incidence rate ratio (95% CI)^b^ |
| Africa |  |  |  |  |  |  |  |
| 1 (lowest) | <0.4 | 34 | 33.8 | 11 | 53.1 | 2.55 (1.60 - 4.05) | 4.62 (2.55 - 8.37) |
| 2 | 0.4 - 0.9 | 73 | 38.6 | 18 | 32.8 | 2.93 (2.20 - 3.90) | 2.76 (1.73 - 4.39) |
| 3 | 0.9 - 1.7 | 41 | 26.1 | 16 | 18.7 | 1.69 (1.15 - 2.49) | 1.24 (0.75 - 2.07) |
| 4 | 1.7 - 3.7 | 54 | 35.8 | 20 | 15.3 | 2.48 (1.80 - 3.42) | 1.07 (0.69 - 1.67) |
| 5 (highest) | 3.7 - 18.5 | 33 | 28.5 | 15 | 18.7 | 1.83 (1.22 - 2.76) | 1.19 (0.71 - 2.00) |
|  |  |  |  |  |  |  |  |
| Europe |  |  |  |  |  |  |  |
| 1 (lowest) | <2.3 | 140 | 18.3 | 52 | 18.6 | 1.92 (1.58 - 2.34) | 2.10 (1.58 - 2.79) |
| 2 | 2.3 - 3.9 | 125 | 15.1 | 69 | 16.5 | 1.61 (1.32 - 1.96) | 1.72 (1.35 - 2.18) |
| 3 | 3.9 - 5.9 | 126 | 17.0 | 88 | 15.8 | 1.50 (1.23 - 1.84) | 1.42 (1.15 - 1.75) |
| 4 | 5.9 - 9.4 | 140 | 20.0 | 102 | 17.5 | 1.89 (1.56 - 2.28) | 1.37 (1.12 - 1.68) |
| 5 (highest) | 9.4 - 26.4 | 54 | 12.8 | 99 | 14.0 | 1.14 (0.85 - 1.53) | 1.17 (0.95 - 1.44) |
|  |  |  |  |  |  |  |  |
| Middle East |  |  |  |  |  |  |  |
| 1 (lowest) | <0.8 | 94 | 24.3 | 13 | 21.5 | 1.42 (1.13 - 1.78) | 1.40 (0.79 - 2.47) |
| 2 | 0.8 - 1.7 | 82 | 19.5 | 19 | 25.7 | 1.10 (0.87 - 1.38) | 1.50 (0.95 - 2.35) |
| 3 | 1.7 - 3.3 | 71 | 19.0 | 20 | 18.4 | 1.04 (0.81 - 1.33) | 0.88 (0.56 - 1.41) |
| 4 | 3.3 - 6.7 | 79 | 24.4 | 29 | 20.3 | 1.28 (1.01 - 1.64) | 1.06 (0.73 - 1.55) |
| 5 (highest) | 6.7 - 40.0 | 84 | 26.3 | 21 | 12.3 | 1.40 (1.08 - 1.81) | 0.58 (0.37 - 0.91) |

^a^The incidence rate measures the number of new cases per 10,000 person years at risk
^b^Adjusted for age, gender, and calendar period the IRR compares rates at each level of ethnic density with the highest ethnic density quintile for that migrant group.
